# Features of Self-stigmatization of Patients with Schizophrenia Spectrum Disorders at the Initial Disease Stage

**DOI:** 10.1192/j.eurpsy.2025.2181

**Published:** 2025-08-26

**Authors:** V. Mitikhin, T. Solokhina, D. Oshevsky

**Affiliations:** 1 Mental Health Research Centre, Moscow, Russian Federation

## Abstract

**Introduction:**

Patients with schizophrenia spectrum disorders (SSD) are the most vulnerable category in terms of formation of self-stigmatization (Rayan A., Aldaieflih M., 2019). However, clinical and psychological features of their self-stigmatization were studied mainly at the longer stages of the disease. Self-stigmatization of patients at the initial stages of the disease rarely got to the attention of researchers, what determined the relevance of the study.

**Objectives:**

To identify characteristics of self-stigmatization patients with SSD at the initial stage of the disease; to determine their needs in psychosocial treatment.

**Methods:**

Questionnaire for assessing the phenomenon of self-stigmatization of mentally ill people (Mikhailova et al., 2005) and PANSS were used. A group of 39 patients (23 women and 16 men) with SSD (F20.ххх, F23.ххх, F25.ххх according to ICD-10) were examined. The average age of the patients was 28.95±8.53 years. The duration of the disorder varied within 0.5-3 years.

**Results:**

Patients at the initial stages of SSD demonstrated relatively low level of self-stigma. The indicator «General level of self-stigma» was slightly lower than the average values and constituted 0.86±0.53 points. Patients believed that mental disorder and associated changes will not limit their education and work (0.80±0.57 points), social activities (0.78±0.49 points) and self-realization (0.60±0.48 points). Rejection to restrictions caused by mental illness, underestimation of possible social and interpersonal problems and desire to distance from people with mental disorders (0.74±0.56 points) were identified. Correlation analysis of the named Questionnaire on self-stigmatization and PANSS scales revealed the strongest correlations across parameters «Readiness to distance oneself from mentally ill people in the sphere of internal activity», characterizing the rejection of changes that have occurred as a result of the disease, with scales: P2 - judgment disorders, G-12 - decreased criticality to one’s condition and N-1 - dullness of affect (r=0.61 at p=0.003, r=0.54 at p = 0.003, r=0.52 at p=0.006, correspondingly). Thus, relatively low level of self-stigmatization of patients with SSD at the initial stage of the disease is associated with insufficiently critical self-assessment of their state, underestimation of possible social and interpersonal problems, and desire to distance from people with mental disorders.

**Conclusions:**

It is necessary to carry out psychoeducation programs for patients with SSD, aimed at developing an adequate perception of mental disorder and prevention of possible consequences of self-stigmatization, as well as social and communication skills’ development trainings.

**Disclosure of Interest:**

None Declared

